# Machine learning approaches reveal highly heterogeneous air quality co-benefits of the energy transition

**DOI:** 10.1016/j.isci.2023.107652

**Published:** 2023-08-18

**Authors:** Da Zhang, Qingyi Wang, Shaojie Song, Simiao Chen, Mingwei Li, Lu Shen, Siqi Zheng, Bofeng Cai, Shenhao Wang, Haotian Zheng

**Affiliations:** 1 Institute of Energy, Economy, and Environment, Tsinghua University, Beijing, China; 2 Joint Program on the Science and Policy of Global Change, Massachusetts Institute of Technology, Cambridge, MA, USA; 3 Department of Civil and Environmental Engineering, Massachusetts Institute of Technology, Cambridge, MA, USA; 4 State Environmental Protection Key Laboratory of Urban Ambient Air Particulate Matter Pollution Prevention and Control & Tianjin Key Laboratory of Urban Transport Emission Research, College of Environmental Science and Engineering, Nankai University, Tianjin 300350, China; 5 CMA-NKU Cooperative Laboratory for Atmospheric Environment Health Research, Tianjin 300350, China; 6 Harvard-China on Energy, Economy, and Environment, Harvard John A. Paulson School of Engineering and Applied Sciences, Harvard University, Cambridge, MA 02138, USA; 7 Heidelberg Institute of Global Health, Faculty of Medicine and University Hospital, Heidelberg University, Heidelberg, Germany; 8 Chinese Academy of Medical Sciences and Peking Union Medical College, Beijing, China; 9 Center for Policy Research on Energy and the Environment, Princeton University, Princeton, NJ, USA; 10 Department of Atmospheric and Oceanic Sciences, School of Physics, Peking University, Beijing, China; 11 Department of Urban Studies and Planning, Massachusetts Institute of Technology, Cambridge, MA, USA; 12 Center for Carbon Neutrality, Chinese Academy of Environmental Planning, Beijing, China; 13 Media Lab, Massachusetts Institute of Technology, Cambridge, MA, USA; 14 State Key Joint Laboratory of Environmental Simulation and Pollution Control, School of Environment, Tsinghua University, Beijing, China; 15 State Environmental Protection Key Laboratory of Sources and Control of Air Pollution Complex, Beijing, China

**Keywords:** Atmospheric science, Atmospheric chemistry, Machine learning, Energy sustainability

## Abstract

Estimating health benefits of reducing fossil fuel use from improved air quality provides important rationales for carbon emissions abatement. Simulating pollution concentration is a crucial step of the estimation, but traditional approaches often rely on complicated chemical transport models that require extensive expertise and computational resources. In this study, we develop a machine learning framework that is able to provide precise and robust annual average fine particle (PM_2.5_) concentration estimations directly from a high-resolution fossil energy use dataset. Applications of the framework with Chinese data reveal highly heterogeneous health benefits of avoiding premature mortality by reducing fossil fuel use in different sectors and regions in China with a mean of $19/tCO_2_ and a standard deviation of $38/tCO_2_. Reducing rural and residential coal use offers the highest co-benefits with a mean of $151/tCO_2_. Our findings prompt careful policy designs to maximize cost-effectiveness in the transition toward a carbon-neutral energy system.

## Highlights

• We use machine learning to predict annual PM_2.5_ concentration from fossil energy use
• Health benefits from reducing fossil energy in China: $19/tCO_2_, mean; $38/tCO_2_, s.d.
• Reducing rural and residential coal use has the highest health co-benefit ($151/tCO_2_)

## Introduction

The use of fossil fuels is well known as the major anthropogenic source of both greenhouse gas (GHG) and air pollutant emissions in most nations.^1^ Emissions abatement from fossil-fuel use reduction therefore could bring significant health benefits while addressing climate change.^2^^,^^3^^,^^4^^,^^5^^,^^6^^,^^7^^,^^8^^,^^9^^,^^10^ Understanding the heterogeneity of local health co-benefits from marginal emissions reductions by energy transition policies is crucial for designing co-control abatement activities. To obtain the geographical distribution of health co-benefits with high resolution, it usually requires adjoint,^11^ or more commonly, reduced-form chemical transport models (CTMs) that can be iterated many times to simulate air quality changes.^12^^,^^13^^,^^14^^,^^15^^,^^16^ These reduced-form air quality models, e.g., the Air Pollution Emission Experiments and Policy analysis model (APEEP)^17^ and its updated versions (AP2/AP3), the Estimating Air quality Social Impacts Using Regression (EASIUR),^18^ the Intervention Model for Air Pollution (InMAP),^19^ and the TM5-Fast Scenario Screening Tool (TM5-FASST),^20^ often rely on linearized representations of emissions-concentration sensitivity derived from full-scale CTMs.

Although reduced-form models provide satisfying approximations for full-scale models, several important limitations restrict their use in policy analysis. First, inputs for the reduced-form model need to be developed from a full-scale model (usually for a specific simulation period) and are still resource-intensive, hence updating the meteorological conditions that dictate the simulation results requires substantial domain knowledge and efforts. Second, for the atmospheric chemistry processes that are less well-understood, the biases embodied in the full-scale model will be inherited by the reduced-form model, hence affecting the accuracy of policy simulations. Third, similar to the full-scale model, the reduced-form model also needs a detailed list of an emissions inventory for all the pollutant species as inputs. To address these limitations, in this paper, we develop a machine-learning framework that is able to provide precise and robust annual average fine particle (PM_2.5_) concentration estimations directly from high-resolution fossil energy use data and additional geographic information. We apply this framework to the data from China and estimate health co-benefits of reducing fossil fuel use in different sectors and regions. The framework is transparent, easy to update, and ready to be extended to other nations without establishing sophisticated full-scale CTMs.

Our framework also supplements the growing literature that applies machine learning approaches to study air-quality-related topics. Several studies have exploited the strong data-fitting capacity of machine learning methods to predict short-term pollutant concentrations. For example, Ong et al.^21^ use a deep recurrent neural network (DRNN) to predict the next 12-h PM_2.5_ concentrations in 52 Japanese cities; Kerckhoffs et al.^22^ compare performances of different machine learning methods (e.g., bagging and random forest) to predict an average of three 24-h measurements of ultrafine particles (UFP) using mobile and short-term stationary measurements in Dutch cities; Xing et al.^23^ develop a deep-learning-based response surface model (DeepRSM) that is trained using the Community Multiscale Air Quality (CMAQ) simulations on domains that cover China to characterize the response of O_3_ and PM_2.5_ concentrations to emissions changes; Kelp et al.^24^ discuss the design of stable, general machine-learned models of the atmospheric chemical system. There are also studies^25^^,^^26^ that utilize machine learning methods, for example, deep belief network (DBN) and generalized regression neural network (GRNN), to predict long-term (seasonal or annual) PM_2.5_ concentrations using aerosol optical depth (AOD) data. Despite these existing studies, our paper applies machine learning approaches to predict long-term air quality using emissions sources data directly (i.e., fossil energy use information) as well as health co-benefits from avoided premature mortality through reducing CO_2_ emissions from fossil energy use for a large country, i.e., China, for the first time, providing highly relevant implications for policy making. In China, seven regional CO_2_ emissions trading scheme (ETS) pilots have been operating since 2013. Their prices have shown significant variation,^27^^,^^28^ which partly reflects heterogeneous co-benefits of CO_2_ emissions reduction recognized by policymakers in different regions.

## Results

### Structure and performance of the machine-learning framework

Our novel machine-learning framework applies modified convolutional neural network architecture (ResCNN) to simulate the energy-related annual average fine particle (PM_2.5_) concentration observed by China’s national-level air-quality monitoring stations in 2015. The year 2015 is chosen because this is the latest year available for the high-resolution fossil energy CO_2_ emissions dataset^29^ used as key inputs in our framework. The architecture of our framework includes a linear component and a CNN component. A residual connection is added between the linear regression results and the final output for better causal interpretability and model stability. Detailed architecture is shown in Figure S1. We collect 1,497 stations with available data in 2015 from China’s national ambient air quality monitoring network. Some monitoring stations are overlapped in the same grid cell at the geographic resolution we use (10km×10km). This resolution (100 ${km}^{2}$) is finer than most regional reduced-formed models (e.g., 1,642 ${km}^{2}$ (median) for AP2,^17^ 1,296 ${km}^{2}$ (median) for EASIUR,^18^ 293 ${km}^{2}$ (population-weighted mean) for InMAP^19^), and comparable to some comprehensive regional CTMs, for example, 144 ${km}^{2}$ for many CTMs for the United States.^15^ We use the most recent year with available data (the year 2015) for the analysis in this paper, but the framework could be easily extended to future years and other regions. Specifically, the model output is the annual average value of hourly PM_2.5_ concentration for each monitoring station collected from China’s National Air Quality Real-time Disclosure Portal.^30^ The portal publishes hourly readings of PM_2.5_ concentration measured by stations in China’s national ambient air quality monitoring network, which has been developed to generate representative PM_2.5_ concentration levels in different regions in China. We use the annual average value because the relationship between PM_2.5_ exposure and premature mortality has been established on a yearly basis by mainstream epidemiology literature.^31^^,^^32^ For stations co-located within the same grid cell, we group them using their average PM_2.5_ concentration as the output variable, leaving 943 data points in the final dataset for our study. Since some sources are not related to energy consumption, including the dust, residential and open burning of biomass. We modify the labels to exclude the influence of these sources based on government guidelines and the results of previous studies,^33^^,^^34^ as detailed in the Supplemental information.

The model inputs for predicting the annual average PM_2.5_ concentration of a specific monitoring station include 2D tensors that contain the geographical distribution of CO_2_ emissions from fossil energy use (by sector and energy type) as well as altitude, temperature, and precipitation information, with a granular resolution (10km×10km) in a large surrounding area centered by the station (610km×610km, comparable to the domain size used for regional air quality modeling.^35^ Therefore, these 2D tensors are essentially 61×61 matrices. We normalize energy use data when constructing 2D tensors to facilitate the training process, see details in STAR Methods. To capture absolute energy consumption levels and agriculture activity levels (proxied by fertilizer use, livestock, and poultry production per unit area) in the surrounding area (610km×610km) of a station, we include these additional variables shown as the linear architecture (1×11 vector) in Figure S1. Further details about data collection and description are available in in Supplemental information.

**Figure 1:**
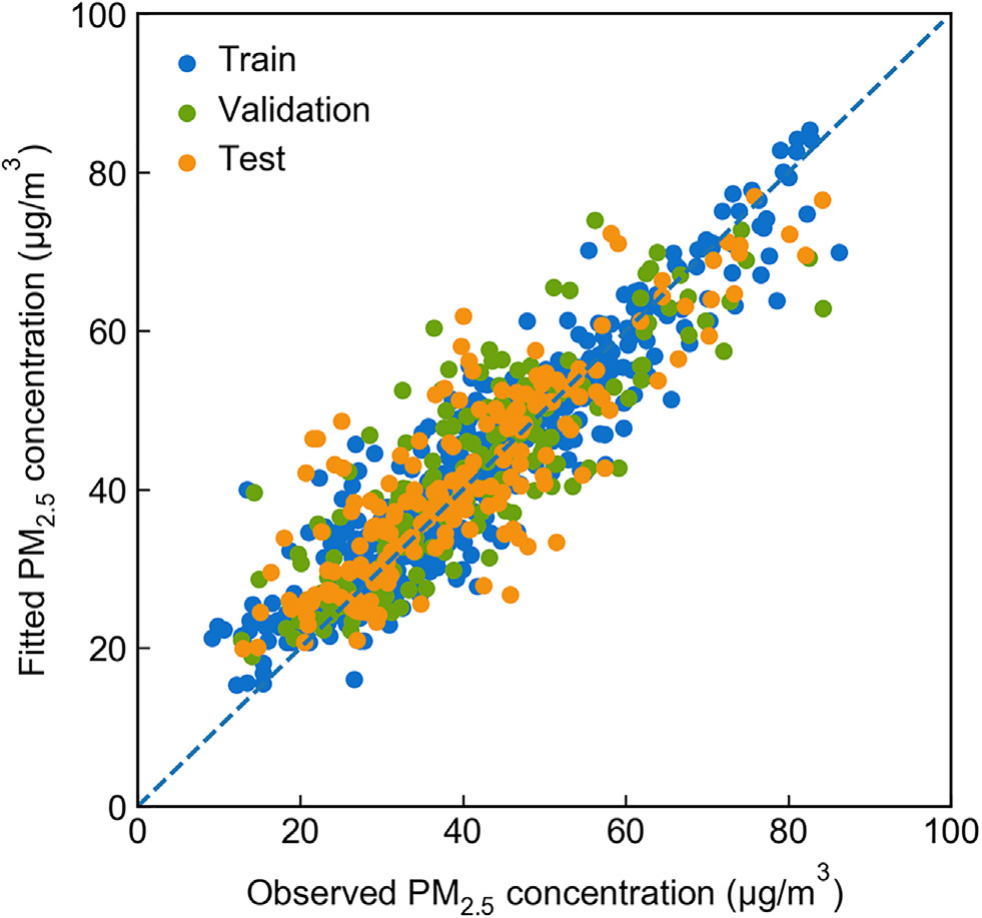
Comparison of model-predicted and observed PM_2.5_ concentrations at 943 stations in mainland China for year 2015 by training, validation, and test datasets

A sequential training procedure is adopted where the coefficient for the hyperparameters for the CNN and the coefficient for the linear component are selected sequentially. The training, validation, and testing set is formed based on stratified sampling by the stations’ geographic regions, and the representativeness of the samples is shown in Table S1. We show the PM_2.5_ prediction performance of our model by comparing the observed and model-predicted concentrations at 943 stations in mainland China for the year 2015 (see Figure 1). The training set shows the best fit, but the validation and test set are also reasonably fitted, suggesting over-fitting is not a major issue. For the test set, mean fractional bias (MFB), mean fractional error (MFE), mean proportional error (MPE), correlation coefficient (ρ), and R square ($R^{2}$) are −0.03, 0.13, 0.14, 0.9, and 0.81, respectively, significantly improved compared to the fit of some published reduced-form air quality models (MFB: −0.06, MFE: 0.36, ρ: 0.74, $R^{2}$: 0.13 in Goodkind et al.^15^ for the contiguous US in 2011; MPE: 0.37, ρ: 0.62 in Muller^17^ for the contiguous US in 2005). This prediction performance is comparable to that of traditional CTMs (MFB: −0.06, MFE: 0.36, $R^{2}$: 0.13 in Tessum et al.^36^ for the contiguous US in 2005; MPB: 0.37, $R^{2}$: 0.13 in Zhang et al. ^37^ for mainland China in 2015), see Table S2. The strong predictive power of our model offers us a considerable advantage over conducting the ensuing air quality co-benefits estimation.

The high predictive performance can be attributed to the innovative design of our ResCNN model, which uses a (λ,1−λ) weighting to combine the benchmark linear regression and the CNN model. By varying the value of λ, we explore the balance between the linear and nonlinear components until identifying the optimum ratio as $\lambda^{*}\approx 0.2$. It implies that only around 20% of the optimum ResCNN structure resembles the classical linear regression, and around 80% of the ResCNN stems from the CNN architecture. The PM_2.5_ pollution is near-linear to the primary PM emission and non-linear to the emissions of gaseous precursors.^38^ We use the combination of the linear part and the CNN based on this prior knowledge to reduce the required training data.^39^ The linear component stabilizes the whole ResCNN system, and the CNN components boost the predictive performance beyond a simple linear benchmark. Compared to the prediction accuracy of a pure linear regression that usually serves as a benchmark of non-machine learning methods (*R*^*2*^: 0.56), a significantly higher accuracy (*R*^*2*^: 0.81) can be achieved by our ResCNN model.

In addition, the linear and non-linear components in the ResCNN architecture take a simple additive form. As a result, the marginal effects of the ResCNN model can be interpreted as causal when the standard conditional independence (CI) assumption holds. The STAR Methods section and the Supplemental information document in detail why the ResCNN outperforms both the reduced-form linear model and the data-driven CNN model and when it can be interpreted as causal. When the standard CI holds, the counterfactual policy analysis can be interpreted as causal. However, as with any causal interpretation using machine learning approaches and observational data only, the causal relationships need to be taken with a grain of salt due to possible confounding variables. Nevertheless, the results from policy scenarios described in the following text at least provide valuable predictive insights into the marginal effects of energy consumption changes.

We simulate annual average PM_2.5_ concentration changes under different policy scenarios with reduced fossil energy use. In this study, we focus on industrial coal use (including coal use in the power sector), road transportation oil use, and rural and residential coal use (mainly for heating and cooking), as they are much discussed in China’s energy and environmental policy making.^37^ For illustration purposes, we first simulate scenarios that curtail fossil energy use in the previous sectors by a certain percentage (from 2% to 20% with a step of 2%), respectively. Note that all emissions reduction simulations are conditional on other variables z. We then estimate population-weighted PM_2.5_ concentration changes and calculate avoided premature deaths in each scenario based on the most recent concentration-response functions. Here, only premature mortality is considered in our health co-benefit estimation as the exposure-mortality relationship is well-established in the epidemiology literature. We do not consider reduced morbidity or labor productivity loss from air quality improvement. We do not include the benefits of avoiding other dimensions of “social costs of carbon”^40^ either.

We find that although the amount of coal use in the industrial sector is about 40 times higher than rural and residential coal use, the deaths avoided by reducing industrial coal use are only about ten times higher, suggesting unit pollutant emissions and marginal air-quality-related health damage (hereafter as health damage) of rural and residential coal use are substantially higher. This result is consistent with the recent finding by Yun et al.^41^ that China’s residential sector contributed only 7.5% of energy consumption but contributed 27% of primary PM_2.5_ emissions and 23% of the outdoor PM_2.5_ concentrations, respectively.

To showcase the computational advantage of our framework, we further downscale the health benefit calculation by estimating marginal health damages of fossil fuel use (per ton of coal equivalent) in these sectors by each grid cell (10km×10km). As expected, there is a wide spread of marginal health damage of fossil fuel consumption across different sectors and within each sector spatially. We find that reducing rural and residential coal use offers the highest health benefits per ton of use reduction. Further information about the framework is provided in STAR Methods.

### Health co-benefits estimations

**Figure 2:**
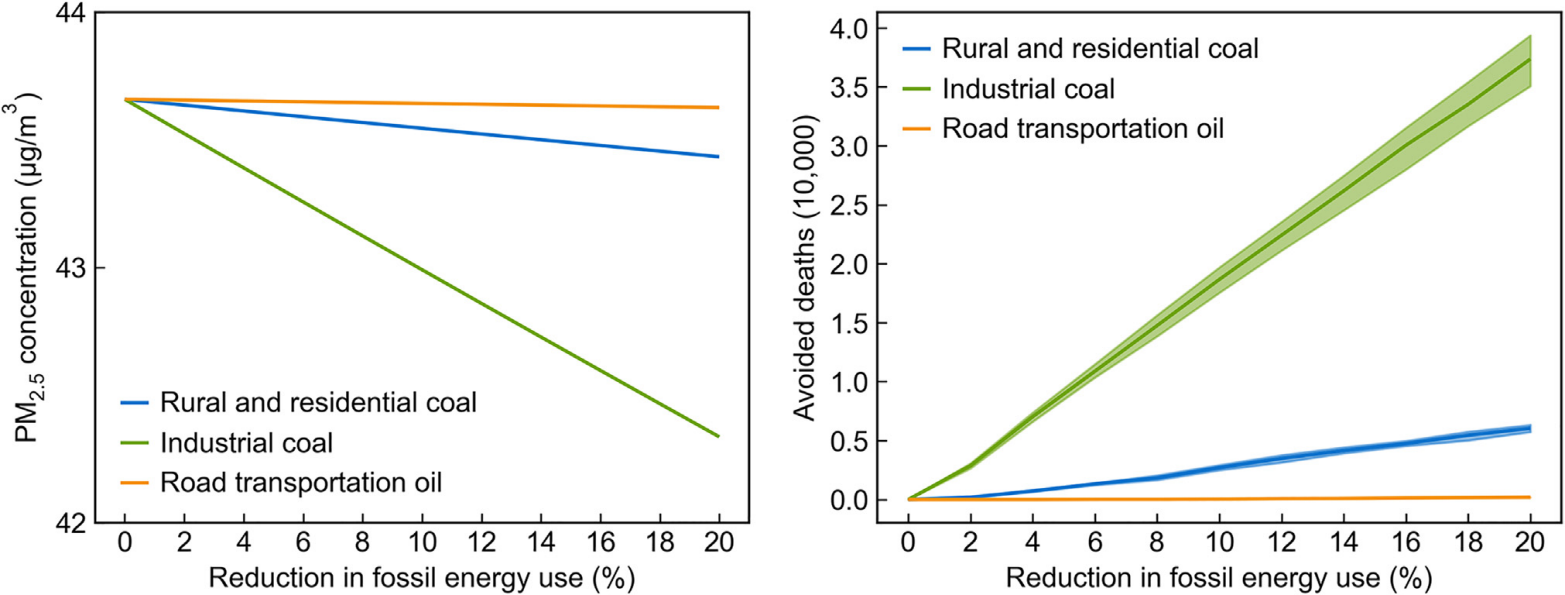
Population-weighted PM_2.5_ concentrations and corresponding avoided deaths (shaded areas represent 90% confidence intervals) in 2015 in mainland China if fossil energy use in one polluting sector were curtailed by a certain percentage (from 2% to 20%)

We first illustrate how our model could be applied in conventional scenario analyses to estimate air quality and health benefits changes with policies aiming to reduce fossil fuel use. We select three key combustion sources in China as our targets, i.e., industrial coal use, rural and residential coal use, and road transportation oil use. Figure 2 shows the national population-weighted PM_2.5_ concentration, and corresponding avoided premature deaths if fossil energy use in one aforementioned sector is curtailed by a certain percentage (from 2% to 20% with a step of 2%), holding other emissions sources and meteorological conditions unchanged. We find the PM_2.5_ concentration decreases, and corresponding avoided deaths increase almost linearly with the size of fossil energy curtailment. Annual avoided deaths from reducing 20% of fossil fuels from these three sectors are 37 thousand, 6 thousand, and 0.2 thousand for industrial coal, rural and residential coal, and road transportation oil, respectively. Avoided deaths from reducing industrial coal are about six times higher than from reducing rural and residential coal use, although CO_2_ emissions (and energy consumption) from industrial coal use are much higher than emissions from the other two sectors (7.4 gigatons for industrial coal, 0.2 gigatons for rural and residential coal, and 1.0 gigaton for road transportation oil). This result reflects the fact that the emissions factor of industrial coal use is significantly lower thanks to strict end-of-pipe measures implemented since the promulgation of the toughest-ever Air Pollution Prevention and Control Action Plan in 2013,^37^ and that locations of emissions sources might be further away from densely populated areas. While most effective air quality control measures promoted by the Action Plan have been targeted at the industrial sector,^37^ we show that there are enormous cost-effective potentials to further harvest health benefits from reducing rural and residential coal use.

**Figure 3:**
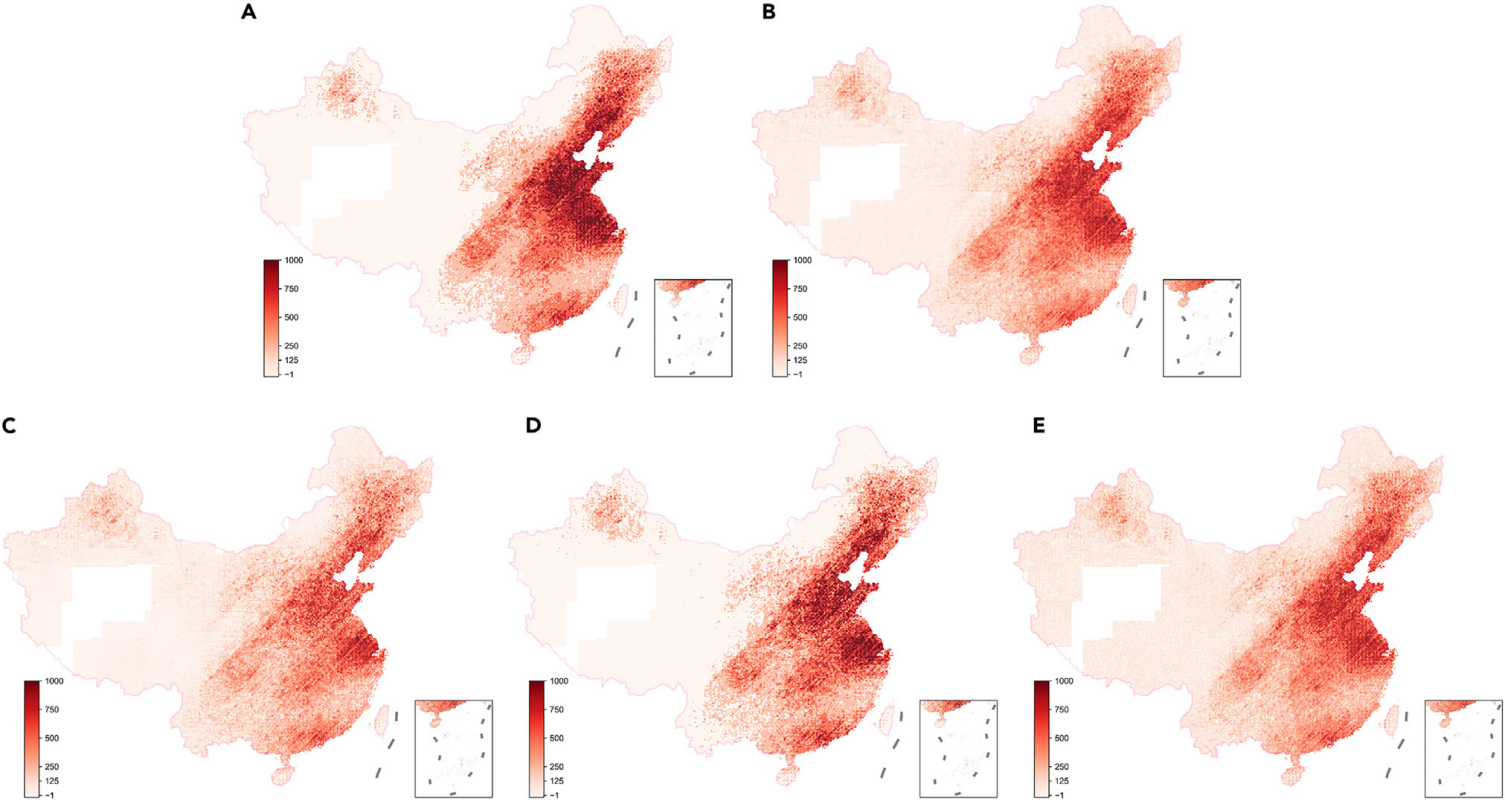
Marginal health damages measured in dollars attributable to an additional ton of CO_2_ emissions from (A) rural and residential coal use, (B) coal use in the industry sector, (C) oil use in the industry sector, (D) coal use in the service sector, and (E) oil use in the transportation sector

We then demonstrate how our model could offer estimated spatial distribution of marginal health damage of fossil energy use in different sectors, a task that will require an unacceptably long simulation time using conventional CTMs. We show marginal health damages attributable to an additional ton of CO_2_ emissions from a certain type of fossil energy use at every source location in mainland China in Figure 3, with maps for five major air-polluting emissions sources considered in our model (i.e., industrial coal use, rural and residential coal use, coal use in the service sector, road transportation oil use, and industrial oil use). We adopt the value of statistical life (VSL) estimate as 1.8 million in US$(2015), see STAR Methods for details. Average marginal health damage per unit ton of CO_2_ emissions (weighted by CO_2_ emissions levels of all the grid cells) ranges from 5 to 151 dollars (US$ 2015 price) for these sectors, with the highest health damages for emissions from rural and residential coal use (151 $/ton) followed by coal use in the service sector (128 $/ton). Health damages for emissions from industrial coal use, road transportation oil, and industrial oil use are one order of magnitude lower (14 $/ton for industrial coal use, 9 $/ton for industrial oil use, and 5 $/ton for road transportation oil use). The mean and standard deviation of marginal health damages attributable to an additional ton of CO_2_ emissions in China are 19 $/ton and 38 $/ton, respectively. The mean value has the same order of magnitude compared to the estimate with a constant VSL of 1.5 million US$(2005) in Vandyck et al.,^9^ although the marginal health damage distribution has a long tail with the 95th percentile value reaching 150 $/ton. Similar to the pattern found in Goodkind et al.,^15^ all the sector-level distributions are positively skewed, indicating high marginal health damages for some hot spots, especially for rural and residential coal use, with its 95th percentile value reaching 281 $/ton (see Figure S2). The large spread across energy use sources and locations reflects substantial differences in coal quality, combustion condition, and end-of-pipe treatment. Overall, the mean marginal health damage of CO_2_ emissions from coal and oil use in China is 21$ and 9$/ton, respectively.

**Figure 4:**
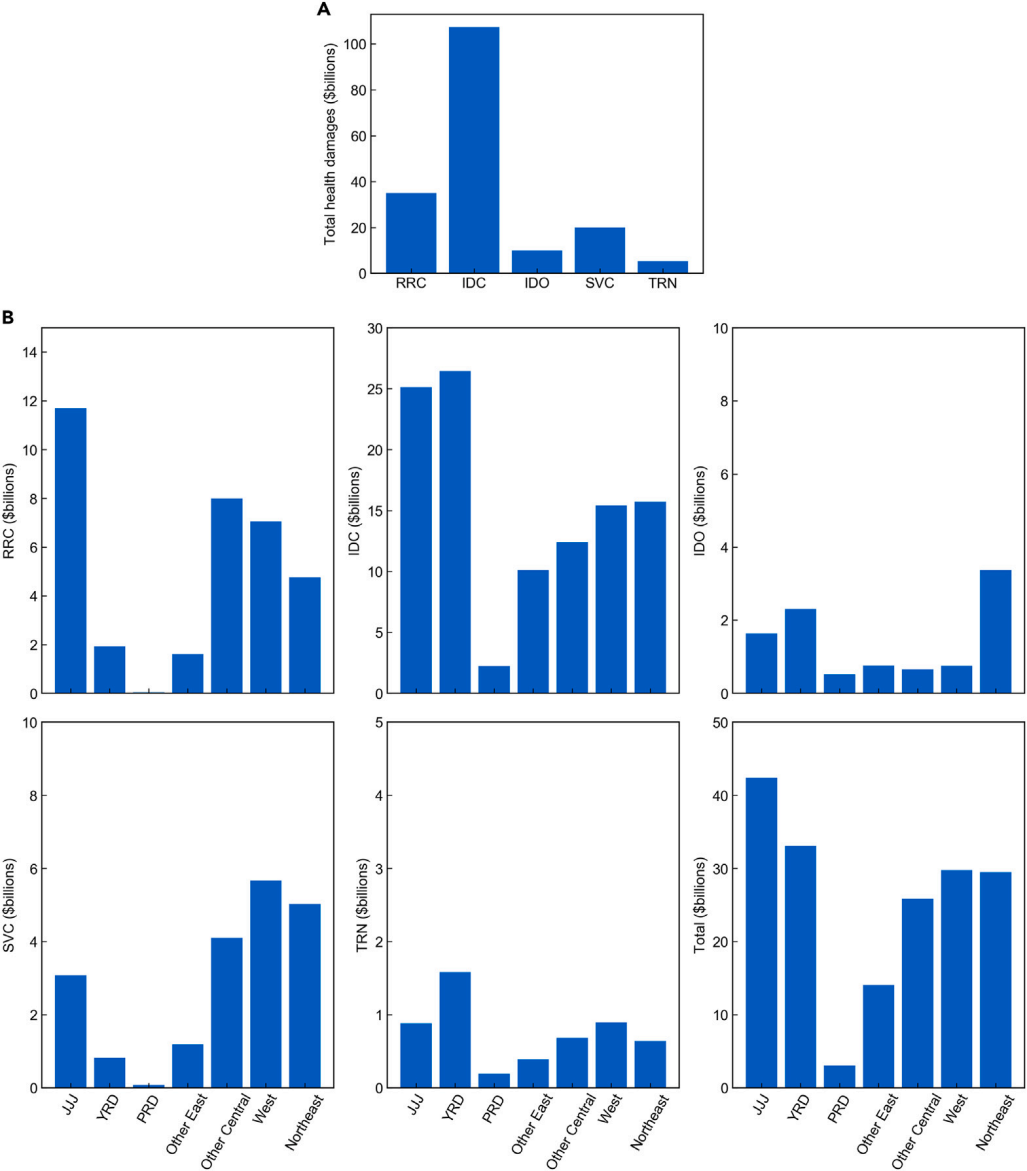
Total health damages from fossil energy use in mainland China (A) by sector and (B) by region with sectoral breakouts RRC: rural and residential coal use; IDC: industrial coal use; IDO: industrial oil use; SVC: coal use in service industry; TRN: oil use in road transportation; JJJ: Bejing-Tianjin-Hebei; YRD: Yangtze River Delta provinces; PRD: Pearl River Delta provinces.

Combining estimated marginal health damages and fossil energy use data with sectoral and regional source information, we can calculate total health damages by sector and by region. We follow the definition of total health damages recommended by Muller et al.,^42^ where the value of pollution damage is the marginal value of pollution times the quantity of pollution. This definition is consistent with the standard conventions of national accounting and has been adopted in multiple empirical studies^15^^,^^16^^,^^43^ to facilitate cross-sector and cross-region comparisons of health damages. For illustration purposes, we aggregate all the mainland Chinese cities into seven regions following the literature^37^^,^^44^ and China’s official document^45^: Bejing-Tianjin-Hebei (JJJ representing the first characters of three provinces’ short names) and surrounding cities (some cities in Shanxi, Shandong, and Henan province included), Yangtze River Delta provinces (YRD), Pearl River Delta provinces (PRD), other East, other Central, West, and Northeast (the first three regions are China’s major air-pollution control regions). Figure 4A shows total health damages by sector. Industrial coal use has the largest total health damage (around $110 billion), while the total health damage of rural and residential coal use is about one-third of the industrial coal’s total health damage. The other types of fossil fuel use have one order of magnitude smaller total health damages. Figure 4B shows total health damages with sectoral breakouts by region. As one of the most populous and polluted regions, the Bejing-Tianjin-Hebei and surrounding cities bear the highest total health damage from fossil fuel emissions (around $40 billion). Its rural and residential coal use imposes substantially higher total health damages than other regions because winter heating contributes much more to the total PM_2.5_ pollution compared to other major air-pollution control regions with less heating demand, e.g., Yangtze River Delta where the total health damage is the second highest (around $30 billion). Some regions show high health damages from a specific type of fossil energy use, e.g., service sector coal use in the Northeast, reflecting the relatively concentrated energy use of this type or associated high marginal health damage in some hot spots in these regions.

The selection of VSL can strongly influence the calculation of the marginal health damage. While we use the estimation of 1.8 million in US$(2015) as an international reference, we also estimate the VSL based on the annual earnings in China. According to Aunan et al.,^46^ the mean VSL is 100 times of annual earnings in China (95% confidence interval, 50 to 150) with a median of 0.8 million in US$(2015). In this case, the marginal health damage would be 56% lower than the aforementioned values. The mean marginal health damage of CO_2_ emissions would be 8$ and 67$/ton for all fossil fuel use and residential coal use, respectively.

### Robustness analysis

We examine the robustness of the emissions-concentration relationship derived from our approach by re-calculating the average marginal health damage using models trained by different sets of hyper-parameters. We select four additional sets of hyper-parameters that produce the smallest weighted least square errors on the validation datasets after the set of hyper-parameters chosen for our base-case model. The mean and standard deviation of marginal health damage of CO_2_ is 20–35 $/ton and 22–48 $/ton, which are close to the results from our base-case model (19 $/ton and 38 $/ton).

We then evaluate if the selection of surrounding area size for the PM_2.5_ monitoring station could affect the prediction accuracy of our framework. Ideally, we should include an area as large as possible to incorporate the impacts of possible long-distance pollutant transportation. In practice, certain thresholds are usually chosen to avoid data availability and computational issues. Besides our base-case model with input tensors representing 610km×610km area centered by a monitor station, we report the prediction accuracy of two alternative models with smaller input tensors (210km×210km and 410km×410km) but identical training processes. Surprisingly, models with smaller input tensors can achieve almost similar accuracy compared to our base-case model on the training dataset. Our base-case model does not show any significant supremacy on the test dataset either, see Figure S3. This result suggests that emissions from a closer surrounding area (210km×210km) dictate the concentration prediction results, and our modeling framework could achieve satisfactory accuracy as long as most relevant input data (fossil energy consumption within a certain distance from the station) are included.

**Figure 5:**
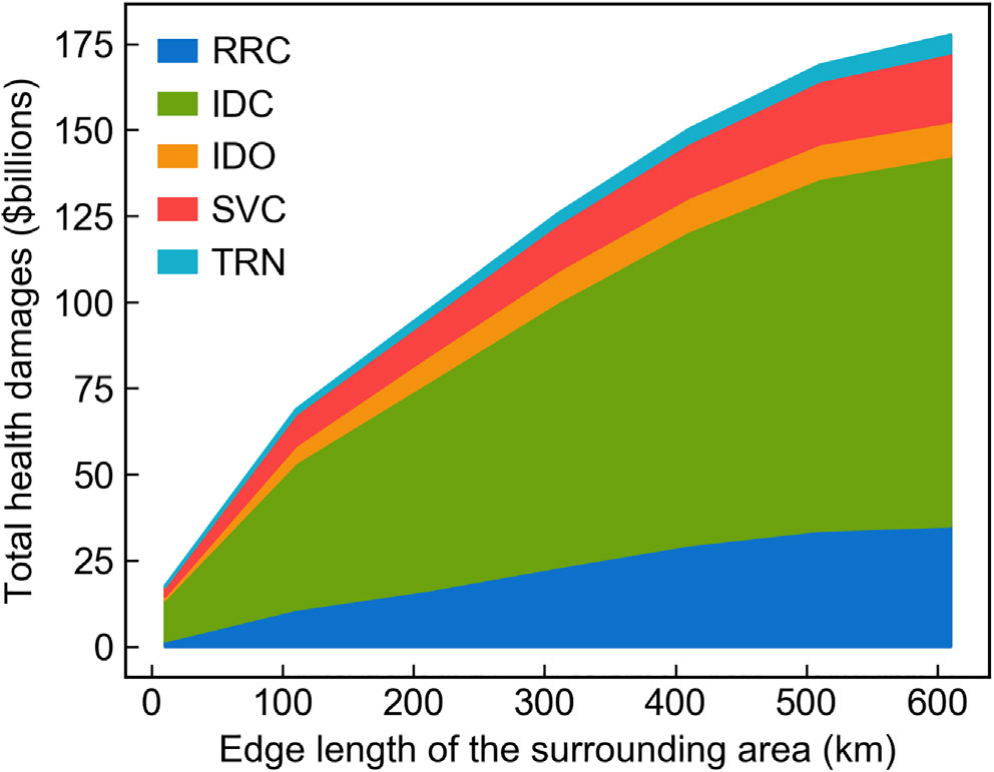
Total health damages by distance (measured by the edge length of the square centered at a monitor station) and emissions source type RRC: rural and residential coal use; IDC: industrial coal use; IDO: industrial oil use; SVC: coal use in service industry; TRN: oil use in road transportation.

To quantify the spatial distribution of health damages caused by pollution sources, we calculate the total health damage within different sizes of a surrounding area centered by monitor stations using our base-case model. Figure 5 shows the total health damage caused by emissions within a certain size of squares (from 110km×110km to 610km×610km) centered by monitor stations. The total health damage increases linearly with regards to the edge length of the surrounding area, indicating a concave relationship with the area size (square of the edge length). Emissions from a faraway source have a smaller impact on the PM_2.5_ concentration of the destination. More than half of the total health damage caused by emissions from the full 610km×610km area occurs within the centering 310km×310km area (one-quarter of the full area). However, compared to the results found by Goodkind et al.^15^ (half of the total PM_2.5_ health damages of a pollution source within a 4,096 km radius circle centered by the source are incurred by people living within 32 km of a source), the total health damage curve in China is less concave, suggesting health damages could be more substantially caused by faraway sources. This different pattern emerges possibly because different distributions of pollution sources and population centers in China allow the atmospheric transmission to form local pollution from more faraway sources.

## Discussion

It is well-recognized that marginal health damage of emissions and corresponding health benefits of abatement vary widely by place and pollution type. Traditional complex CTMs are not suitable for economic and policy analyses that require many model iterations to explore the variation of marginal health damages. Recent developments of reduced-form models with satisfactory approximations for full-scale models have allowed researchers to quantify the heterogeneity of the marginal health damage but still place some limitations on broader applications. In this study, we develop a machine learning-based framework that can produce more accurate, accessible, and easy-to-update simulations for air quality, or more specifically, PM_2.5_ concentrations. Applying this framework to estimate the marginal health damage of CO_2_ emissions by fossil fuel type and source location in China provides us with important insights for China’s future policy designs that aim to achieve ambitious air quality and climate targets.

We use the ambient PM_2.5_ concentration as labels to build the model and estimate the influence of each sector. However, residential fossil fuels not only contribute to ambient air pollution but also significantly affect indoor air pollution. Previous studies^33^ estimated that household solid fuels contributed 43% to the total PM_2.5_-related mortality considering both ambient and indoor air pollution in China in 2015. Therefore, the marginal health damage could be significantly higher than current results when indoor air pollution is considered.

We find that China’s current carbon pricing stringency does not match the magnitude of marginal health damage. Although many command-and-control climate policies are in place in China, limited market-based instruments have been applied. By far, China’s recently launched national CO_2_ ETS only covers the power generation sector, with a mild carbon price at the magnitude of 8 $/ton, substantially less than the average marginal health damage (19 $/ton) estimated by this study. Since this health damage only accounts for premature mortality, this policy gap would be more salient if co-benefits from reduced morbidity or labor productivity loss from air quality improvement and from avoiding other dimensions of “social costs of carbon” were considered. We urge a more accelerated development of China’s national ETS to form an appropriate carbon price that can better internalize the public health externality of CO_2_ emissions.

The highly heterogeneous marginal health damage by fuel type and location also has important implications. By far, only CO_2_ emissions from industrial fossil fuel use are planned to be covered by China’s national ETS^47^ Our analysis shows that, however, CO_2_ emissions from industrial fossil energy use have much lower marginal health damages compared to the emissions from coal use in the rural and residential as well as service sectors. Therefore, complementary policies that could encourage fuel switching (e.g., coal to natural gas for small boilers or electrification of residential heating) in these sectors are essential. Placing an effective carbon price to the sectoral-average level of marginal health damage could be one option, then reductions in fossil fuel use in these sectors would be determined by these sectoral-specific carbon prices and heterogeneous marginal abatement cost curves. However, fuel cost increases caused by carbon pricing would usually impose the highest burdens on low-income households.^48^ To ensure environmental justice while promoting the clean energy transition and addressing climate change^14^^,^^49^^,^^50^^,^^51^ policymakers may consider more progressive policy instruments, such as clean fuel subsidies or clean infrastructure investment. We also note that the marginal health damage of CO_2_ emissions in more populous eastern China, particularly in the North China Plain and Yangtze River Delta region, is much higher than that of emissions in the rest of the country, suggesting a trading ratio^52^ could be considered in the future design of China’s national ETS.

**Figure 6:**
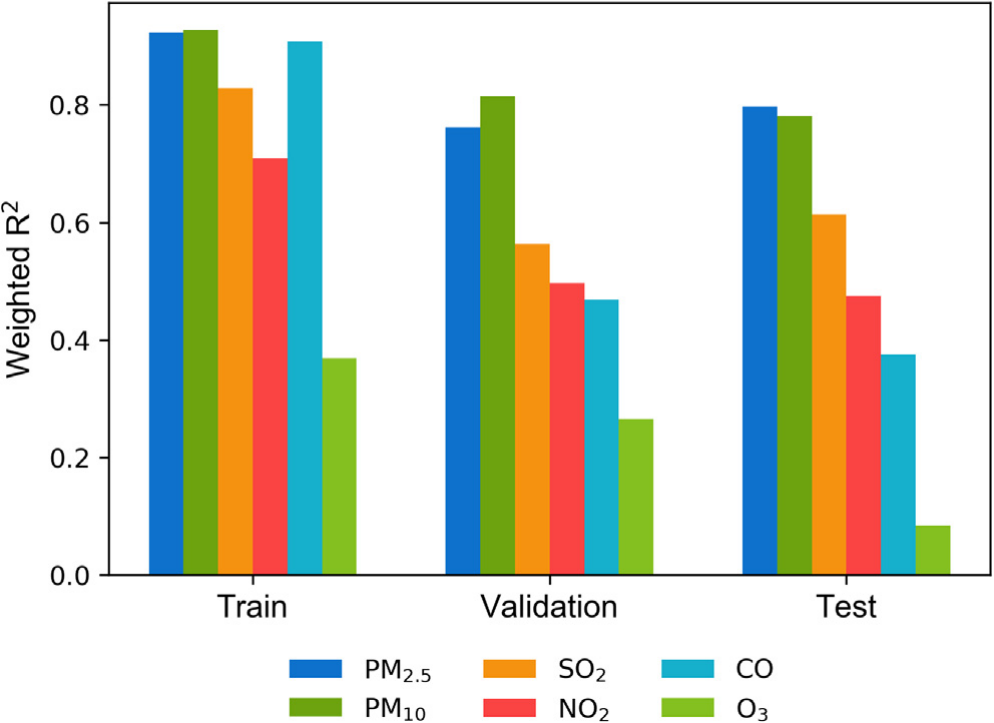
Prediction accuracy (measured by weighted $R^{2}$ on the train, validation, and test datasets) by model with output variables for different pollutant concentrations

Applying machine learning techniques in the environmental integrated assessment motivates fruitful future work. Although our framework provides satisfactory results for the annual PM_2.5_ concentration estimation, prediction accuracy for pollutants other than particular matters needs further improvement. Figure 6 shows the prediction accuracy for PM_10_, SO_2_, NO_2_, CO, and O_3_ by applying the same framework and training process to these pollutants. The accuracy of PM_10_ is similar to that of PM_2.5_. For SO_2_, NO_2_, and CO, the framework can achieve acceptable fitting results on the training dataset but fail to achieve consistent accuracy on the validation and test datasets; the fitness is low even on the training dataset for ozone. Refining the framework architecture and training process and incorporating relevant atmospheric condition data for pollutants other than particular matters remains an interesting direction for future research.

In addition to updating the analysis when input data are available for more recent years, we believe it is also important to extend the co-benefits analysis implemented in this study to more developing countries with imperative air pollution control and climate mitigation policy development. High-resolution marginal health damage estimations that are very relevant for policy making have been almost exclusively conducted in the United States,^10^^,^^11^^,^^12^^,^^13^^,^^14^^,^^15^ suggesting the complexity of the methodology even with elaborately designed reduced-form CTMs. Our machine learning framework can supply a more accessible tool for researchers and policymakers to implement more comprehensive and timely analyses in developing countries that are constrained by conventional emissions inventory and atmospheric measurement data, as new high-resolved energy consumption datasets^53^^,^^54^ become more readily available.

### Limitations of the study

Several caveats should be noted. (i) We acknowledge that even though we have included as many related variables as possible in the model and deducted the contribution of non-energy-related sources from the labels, the CI assumption may still not be strong enough to hold as there could be additional confounding factors and unobserved confounders that we cannot take into consideration. With increasingly available data, the causal interpretability and model stability can be improved in future studies. Nevertheless, there could still be some spatially varying unobserved confounders that would undermine the CI assumption and the claim of causality as our model learns the mapping from emissions to concentrations based on their spatial variation. (ii) The end-of-pipe control measures, which can strongly affect the marginal damages of the power and industrial sectors, are not explicitly represented in the model. Future studies can incorporate the influence of control measures in the modeling framework by re-training it with multiple-year data. (iii) The annual average meteorological parameters considered in the current framework may not be enough to fully capture the correlation (potentially spurious) between energy consumption and PM_2.5_ pollution, which are both affected by atmospheric conditions. For instance, the effect of high precipitations when emissions are high may be masked by low precipitations when emissions are low. We tried to integrate monthly or seasonal meteorological data into our model without adjusting its main structure, however, the prediction power did not improve. We believe incorporating more detailed meteorological condition information would require deeper and wider CNNs or alternative models for enhancing the performance, which are beyond the scope of this study. Future studies with more extensive meteorological data and larger training datasets are needed to investigate the impact of these factors on PM_2.5_ pollution prediction.

## STAR★Methods

### Key resources table

**Table:** 

REAGENT or RESOURCE	SOURCE	IDENTIFIER
**Software and algorithms**		

The high-resolution fossil energy CO_2_ emissions dataset	Cai et al.^29^	https://doi.org/10.1016/j.resconrec.2017.10.036
ResCNN Model	This paper	https://github.com/sunnyqywang/cnn_pollution
Python version 3.11	Python Software Foundation	https://www.python.org

### Resource availability

#### Lead contact

Further information and requests for resources and reagents should be directed to and will be fulfilled by the lead contact, Da Zhang (zhangda@tsinghua.edu.cn).

#### Materials availability

This study did not generate new unique reagents.

### Method details

#### Datasets and preprocessing

We denote the inputs used to predict annual average fine particle (PM_2.5_) concentration as $x_{ijkn}\in R^{4}$ with four indices: n is the index of air-quality monitoring stations (1≤n≤N); k is the index of different types of data inputs that are used for prediction, including the level of fossil energy use of a specific energy type in a specific sector as well as some additional geographic information, for example, altitude, temperature, and precipitation (1≤k≤K); and (i,j) is the location index representing a grid cell of an area centered around the station (1≤i,j≤I,J). Therefore, $x_{ijkn}$ represents the value of a specific input k for a grid cell (i,j) surrounding a specific station n. The total number of stations is N=943, and eight (K=8) types of data inputs with the high geographic resolution are used: (a) CO_2_ emissions from rural and residential coal use (RRC), (b) CO_2_ emissions from industrial coal use (IDC), (c) CO_2_ emissions from industrial oil use (IDO), (d) CO_2_ emissions from coal use in the service industry (SVC), (e) CO_2_ emissions from oil use in road transportation (TRN), (f) altitude (ALT), (g) annual average temperature (TEM), and (h) annual average precipitation (PCP). The number of pixels in each 2D tensor in our main model specification is I×J=61×61. With each pixel representing a 10km×10km cell, the geographical coverage of each 2D tensor equals to [I×10]×[J×10]=610km×610km. The large geographical coverage of each station, along with 943 stations in total, guarantees that our analysis covers all the territory of mainland China with permanent residents. We also use additional station-level variables to incorporate the effects of agriculture activity levels, proxied by fertilizer use, livestock, and poultry production per unit area of the city where the station locates.

The output of our main model is the annual average PM_2.5_ concentration, denoted as $y_{n}$. Note that each $y_{n}\in R$ is a real number for each station, different from the high dimensionality of the input variable $x_{ijkn}$. We also train separate models to predict annual average concentrations of other air pollutants, including the annual average concentration of PM_2.5_, PM_10_, SO_2_, NO_2_, CO, and O_3_. While outputs of the models are different, the model structure and the training process are similar to our main model specification.

Inputs $x_{ijkn}$ are normalized before modeling to improve prediction accuracy and facilitate the training process.^55^ The input $x_{ijkn}$ is normalized as following:

(Equation 1) $$x_{ijkn}\leftarrow \frac{x_{ijkn}}{10^{m_{k}}}$$

where $m_{k}$ defines the magnitude of each input type k:

(Equation 2) $$m_{k}=\left\lfloor \log_{10}\left(\max_{i,j,n}x_{ijkn}\right)\right\rfloor$$

For stations that are close to national boundaries or the sea, the 2D tensors could have cells with no energy use information. These cells are filled with zeros.

#### ResCNN model

As visualized in Figure S1, our model architecture consists of two parts. The first part is a standard AlexNet,^56^ which uses the normalized 3D tensor $x_{ijkn}$ as input for each station n. It starts with repeated blocks of convolutional layers and max pooling layers, and ends with several fully connected layers. The second part has a linear specification and average value of each 1D tensor ${\bar{x}}_{kn}$ as input. These two parts provide complementary information: AlexNet absorbs the nonlinear structural spatial information of each 3D tensor, while the linear part absorbs the magnitude information of each 3D tensor, which is lost in AlexNet due to normalization. Intuitively, PM_2.5_ concentration varies with the average amount of the energy use (e.g., higher fossil energy use usually causes higher pollution concentration), and it also depends on some spatial structural information that is missing in the average values (e.g., fossil energy use that is closer to the station will have a larger effect on the concentration, and the spatial distribution of one type of fossil energy use may have a nonlinear interaction with the spatial distribution of another type of fossil energy use). Different from the traditional process of explicitly specifying the nonlinear relationship, our model automatically learns the nonlinear relationship between fossil energy consumption, atmospheric conditions, geographic information, and the resulting annual average concentration level of PM_2.5_. The synthesis of the AlexNet and the linear parts is similar to ResNet,^57^ which is also a linear combination of linear and nonlinear feature maps. We therefore name the model architecture as ResCNN because it combines linear regression to capture the magnitude effect and CNN to capture the residual nonlinear relationship.

The ResCNN model is more general than ResNet,^57^ since it is a weighted average of the linear and the CNN components:

(Equation 3) $$y_{n}=\lambda \beta^{\prime }\left[z_{n},{\bar{x}}_{kn}\right]+\left(1-\lambda \right)f_{c}\left(x_{ijkn}\right)+\epsilon_{n}$$

Equation 3 consists of the linear component $\beta^{\prime }\left[z_{n},{\bar{x}}_{kn}\right]$ and the nonlinear (CNN) component $f_{c}\left(x_{ijkn}\right)$, weighted by the hyper-parameter (λ,1−λ). The β parameters represent the coefficients of the explanatory variables in the linear model to improve interpretability and stability. The λ term is a hyper-parameter that adjusts the ratio of the linear and nonlinear components in the ResCNN. As λ∈[0,1], the λ value can measure the closeness of the ResCNN to a linear model. When λ→1, the ResCNN resembles a linear regression. When λ→0, the ResCNN resembles the CNN model. The two boundary cases of the ResCNN model are the reduced-form linear model and the CNN model, corresponding to the cases in which λ=1 and λ=0. Since 1−λ shrinks the magnitude of the CNN, it resembles the nature of the L2 regularization (Ridge regression), thus mitigating the overfitting of the CNN component. In Equation 3, x represents the treatment variables (CO_2_ emissions from fossil energy use); z represents the control variables; The $\epsilon_{n}$ term is commonly assumed to be the Gaussian noise, as shown in the training process with mean squared error in the objective function. Overall, the ResCNN is a general model family with effective regularization controls, so it can achieve higher predictive performance than both the reduced-form linear model and the standard CNN model. The superior predictive performance of the ResCNN can be found in Section 6.

#### Causal interpretation

The ResCNN is causal when the conditional independence (CI) assumption is satisfied. Although the CNN architecture $f_{c}$ is highly nonlinear, causality exists in the ResCNN model because the three major components of the control variables $z_{n}$, treatment variables $x_{n}$, and random noise $\epsilon_{n}$ take an additive form in Equation 3. Combining the additive structure of ResCNN and the CI assumption, the marginal effect of the ResCNN has a causal interpretation. For further details, please refer to Section 4 in Supplemental information.

The key assumption for the causality of the ResCNN is the independence between the noise term $\epsilon_{n}$ and the treatment $x_{n}$ conditioning on $z_{n}$. This CI assumption is standard for the causal interpretation in linear regression.^58^^,^^59^ In other words, it does not require more assumptions than a linear model for the ResCNN model to obtain causal interpretation. The ResCNN model differs from the linear regression only in the method of estimating the average treatment effect. The ResCNN model uses the nonlinear marginal effect, rather than the simple linear parameters.^58^

Empirically, the CI assumption $x_{n}⊥\epsilon_{n}|z_{n}$ is still challenging to hold. It is because this assumption implies that the major contributors to and the potential confounding factors of PM_2.5_ are all incorporated into our model. We have followed the tradition of air quality modeling and include the variables that can approximate the major primary and secondary sources for PM_2.5_ formation, e.g., fossil energy consumption, agricultural activities, and geographic information. Some omitted factors may still exist, such as volatile organic compounds emissions. However, the dominant factors that affect annual PM_2.5_ concentration levels have already been included in the model to ensure a consistent estimation of the treatment effect.

#### Evaluation metrics

To evaluate the performance of the ResCNN, we have adopted some common evaluation metrics for the performance of the CTMs, for example, coefficient of determination ($R^{2}$), normalized mean bias (NMB), and normalized mean error/mean absolute percentage error (NME/MAPE), from the literature.^17^^,^^19^^,^^20^^,^^60^^,^^61^ We add an important modification to all of the evaluation metrics by introducing normalized weights $w_{n}$ that are proportional to the population that a specific station n corresponds to. For example, if there is only one station that enters our sample for a specific city, we assume the station generates the concentration reading that represents the exposure of the city’s whole population; however, if multiple stations are present in our sample for a city, we assume each station generates the concentration reading that represents an equal share of the city’s population. With this modification, the performance measure gives more weight to the prediction accuracy of stations corresponding to a larger population because the marginal health damage we estimate later is proportional to the population. We illustrate all the metrics we compute in Table S2. Our main model uses the weighted least square errors on the validation set in hyper-parameter searching, which we discuss in the section below.

#### Hyper-parameter searching and training

There are two sets of hyper-parameters: the set of hyper-parameters of CNN and the coefficient of the linear part λ. Since the size of the CNN network is huge compared to the single parameter λ. A sequential training is adopted instead of joint training: a hyper-parameter set is chosen for the CNN part (setting λ=0 and then with the CNN hyperparameters fixed, a search over λ values are conducted for the final model specification.

To address the challenge of specifying the appropriate CNN hyper-parameters, this study combines random search and grid search. First, random searches (400 trials) in a pre-specified, larger hyper-parameter space are done. Conditioning on each hyper-parameter and while keeping the others random, hyper-parameter values that yield consistently lower performance or higher variance in performance are pruned. Then a complete grid search was done to the remaining hyper-parameter values (384 combinations). While numerous methods can be used to identify the best hyper-parameter, the simple random search^62^ is still a useful benchmark, even in comparison to more complicated hyper-parameter searching methods based on reinforcement learning or Gaussian process.^63^^,^^64^^,^^65^ The full hyper-parameter space is shown in Table S3, and the pruned hyper-parameter space is shown in Table S4. Among the hyper-parameters, some are model specific and require some searching in each specific application. For instance, dropout and batch normalization were found as effective regularization in several studies.^66^^,^^67^ Data augmentation^68^ assumes the invariance property: when images are rotated or flipped, the new images should not change the predicted values $\hat{y}$. On the other hand, some hyper-parameters are set to follow the common practice. For instance, Rectified Linear Unit (ReLU) is used as activation functions for each neuron; He initialization^69^ is used to address the problem of vanishing and exploding gradients; Adam optimizer^70^ is used for gradient descent optimization.

The ResCNN model is trained by empirical risk minimization (ERM). Formally,

(Equation 4) $$\min_{W}E\left(W;W_{h}\right)=\min_{W}\frac{1}{N}\sum_{n=1}^{N}w_{n}\times {\left(y_{n}-f\left(z_{n},x_{ijkn};W,W_{h}\right)\right)}^{2}$$

in which W represents parameters and $W_{h}$ represents hyper-parameters, while $w_{n}$ represents the weight of each observation as we describe in the above section. Note that the training in Equation 4 is conditioning on the specific choice of hyper-parameters $W_{h}$. Denote $W^{*}=\underset{W}{\mathrm{argmin}}E\left(W;W_{h}\right)$, the optimum hyper-parameter $W_{h}$ is chosen by random searching:

(Equation 5) $$W_{h}^{*}=\underset{W_{h}\in \left\{W_{h}^{\left(1\right)},W_{h}^{\left(2\right)},\cdots ,W_{h}^{\left(S\right)}\right\}}{\mathrm{argmin}}E\left(W^{*};W_{h}\right)$$

$W_{h}^{\left(S\right)}$ represents a random sample from the hyper-parameter space. The best hyper-parameter $W_{h}^{*}$ is chosen out of S=100 training. To train model and choose hyper-parameters, the full dataset is split into training, validation, and testing sets with a ratio equal to 3:1:1 with a stratified sampling approach. The training set is used to train ResCNN model as in (4); the validation set is used for the selection of hyper-parameter as in Equation 5; the testing set is used for model evaluation and comparison. Further details of the experiment design can be found in Supplemental information Section 5.

#### Health impacts and valuation

We apply the up-to-date GEMM NCD+LRI method^32^ to estimate avoided premature death related to reductions in chronic exposure to outdoor fine particulate matter (PM_2.5_) under different scenarios. The GEMM NCD+LRI method is considered as a major update to the widely-used IER 5-COD approaches^31^^,^^71^ and has been adopted in some recent cost-benefit analysis.^11^^,^^37^^,^^44^ Compared to the IER approach, the GEMM NCD+LRI method incorporates findings from recent cohort studies that quantify the relationship between avoided premature death and ambient PM_2.5_ concentration (C-R relationship) in regions with high PM_2.5_ concentration levels, making it more appropriate to be applied in relatively more polluted countries like China. In addition, the GEMM NCD+LRI method associates PM_2.5_-related death with non-accidental deaths caused by non-communicable diseases and lower respiratory infections, a more comprehensive range than the five specific causes of death considered in the IER 5-COD approaches.

The GEMM NCD+LRI quantifies the relationship between the hazard ratio (RR) of NCD+LRI and ambient PM_2.5_ exposure (c, equal to PM_2.5_ concentration for each grid cell) with the following equation:

(Equation 6) $$RR\left(c\right)=\exp\left(\theta \times \frac{\ln\left(\frac{\max\left(0,c-c_{f}\right)}{\alpha }+1\right)}{1+\exp\left(-\frac{\max\left(0,c-c_{f}\right)-\mu }{\nu }\right)}\right)$$

where θ, α, μ, ν and $c_{f}$ are all shape parameters that define the C-R relationship. Since the baseline mortality rate is different for adults of different ages, we follow the convention to divide the population in a specific grid cell n into 12 subgroups (adults with ages from 25 to 85 and above in five-year intervals). Consider two scenarios (scenario 0 and scenario 1) with different ambient PM_2.5_ concentration levels ($c_{0}$, $c_{1}$), the avoided death under scenario 1 compared to scenario 0 in grid cell n is calculated by summing up avoided deaths of all m age groups:

(Equation 7) $$\Delta M_{n}=\sum_{m}M_{m}^{B}\times {pop}_{m,n}\times \left(\frac{1}{RR\left(c_{1},n\right)}-\frac{1}{RR\left(c_{0},n\right)}\right)$$

where $M_{m}^{B}$ is the baseline mortality rate for age group m in China, retrieved from the Global Health Data Exchange, and ${pop}_{m,n}$ is the population of age group m in grid cell n.

We adopt the assumption that θ has a normal distribution with mean $\mu_{\theta }$ and standard deviation $\sigma_{\theta }\left(50\right)$.^31^ We can then sample 1,000 points from the normal distribution and calculate the mean and 95% confidence interval of avoided death using Equation 7. An alternative, faster approach to obtain the mean avoided death $\bar{\Delta M_{n}}$ is to use the following equation to first calculate the mean of $1/RR\left(c_{n}\right)$, which has a lognormal distribution,

(Equation 8) $$\bar{1/RR\left(c_{n}\right)}\&=\exp\left(-\mu_{\theta }\frac{\ln\left(\frac{\max\left(0,c_{n}-c_{f}\right)}{\alpha }+1\right)}{1+\exp\left(-\frac{\max\left(0,c_{n}-c_{f}\right)-\mu }{\nu }\right)}+\frac{1}{2}{\left(\sigma_{\theta }\frac{\ln\left(\frac{\max\left(0,c_{n}-c_{f}\right)}{\alpha }+1\right)}{1+\exp\left(-\frac{\max\left(0,c_{n}-c_{f}\right)-\mu }{\nu }\right)}\right)}^{2}\right)$$

and then calculate the mean avoided death $\bar{\Delta M_{n}}$.

(Equation 9) $$\bar{\Delta M_{n}}=\sum_{m}M_{m}^{B}\times {pop}_{m,n}\times \left(\bar{1/RR\left(c_{1,n}\right)}-\bar{1/RR\left(c_{0,n}\right)}\right)$$

In our scenario analysis, where sector $k^{\prime }$ emissions are curtailed by p (p ranges from 2% to 20%), $c_{0,n}$ is taken to be the predicted baseline PM_2.5_ concentration, and $c_{1,n}$ is obtained by reducing all input emissions in sector $k^{\prime }$ by p:

(Equation 10) $$c_{1,n}=f\left(x_{ijkn,k\neq k^{\prime }},x_{ijk^{\prime }n}\times \left(1-p\right);W^{*},W_{h}^{*}\right)$$

The marginal change of PM_2.5_ due to the marginal change of emissions in each sector in each 10km×10km cell can be obtained from the gradients of PM_2.5_ with respect to the input emissions from each sector and each grid cell ($\frac{\partial y}{\partial x_{ijk}}$). Since back-propagation is used for training the neural network, the gradients can be directly exported from the models. For each unit of emissions increase in sector $k^{\prime }$ in cell (i,j), the mean avoided death for grid cell n is:

(Equation 11) $$\bar{\Delta M_{ijk^{\prime }n}}=\sum_{m}M_{m}^{B}\times {pop}_{m,n}\times \left[\bar{1/RR\left(c_{0,n}+\frac{\partial y_{n}}{\partial x_{ijk^{\prime }n}}\right)}-\bar{1/RR\left(c_{0},n\right)}\right]$$

where again $c_{0,n}$ is taken to be the predicted baseline PM_2.5_ concentration.

We close the estimate of marginal monetary health damages related to increased premature mortality due to an additional ton of CO_2_ emissions in each sector in each cell by using an inferred value of statistical life (VSL) for China based on the U.S. EPA recommended VSL of 8.7 million in US$(2015).^72^ We adopt the income elasticity for high-income countries recommended by a recent meta-study^73^ for extrapolating from the U.S. VSL (${VSL}_{base}$) to the VSL for China (VSL):

(Equation 12) $$VSL={VSL}_{base}\times {\left({pcGDP}_{China}/{pcGDP}_{U.S.}\right)}^{0.8}$$

where ${pcGDP}_{China}$ and ${pcGDP}_{U.S.}$ represent GDP per capita in 2015 for China and the U.S., respectively. Equation 12 gives the VSL estimate as 1.8 million in US$(2015) for China.

Combining the considerations of premature mortality and the inferred value of statistical life, the marginal monetary health damages due to an additional ton of CO_2_ emissions in sector $k^{\prime }$ in cell (i,j) is

(Equation 13) $$MD_{ijk^{\prime }}=-\sum_{n}VSL\times \bar{\Delta M_{ijk^{\prime }n}}$$

The total monetary health damages incurred by emissions from sector $k^{\prime }$ are the sum of the production of marginal monetary health damages and emissions in each cell:

(Equation 14) $$TD_{k^{\prime }}=\sum_{i,j}MD_{ijk^{\prime }}\times x_{ijk^{\prime }}$$

### Quantification and statistical analysis

Statistical analyses were performed using Python.

## Data Availability

Data and scripts have been uploaded to Github (https://github.com/sunnyqywang/cnn_pollution).
