# Supplementary material for: Machine learning approaches reveal highly heterogeneous air quality co-benefits of the energy transition
Source: iScience. 2023 Aug 18;26(9):107652. doi: 10.1016/j.isci.2023.107652 (PMC10480617; doi:10.1016/j.isci.2023.107652)
Supplement: Document S1. Figures S1–S4 and Tables S1–S4 [file mmc1.pdf]

iScience, Volume *26*

Supplemental information

Machine learning approaches reveal highly heterogeneous air quality co-benefits of the energy transition

Da Zhang, Qingyi Wang, Shaojie Song, Simiao Chen, Mingwei Li, Lu Shen, Siqi Zheng, Bofeng Cai, Shenhao Wang, and Haotian Zheng

Supplementary Text

Data collection and imputation

Table S1 presents four categories of variables in the data set: CO_2 emissions from fossil energy consumption, geographical factors, agricultural factors, and air pollutant concentrations. They are collected from three major sources. CO_2 emissions from fossil energy consumption and the geographic information with a high spatial resolution (values available by $10\ \mathrm{km} \times 10\ \mathrm{km}$ grid cells) are provided by the China high-resolution emissions database (CHRED) data set, the details of which can be found in related works[69]. The air pollution data, including the $PM_{2.5}$ concentration, is collected from China's National Air Quality Real-time Disclosure Portal. The network for collecting the air pollution data includes 1,532 monitoring stations, among which 1,497 stations' data is incorporated into the present study. The 35 (=1,532-1,497) stations are excluded since they are either newly built or malfunctioning such that the year-round $PM_{2.5}$ data is incomplete. The agricultural data is augmented to our data set from the municipal statistical yearbooks.

The raw data is of high quality since the 1,497 data points do not contain much missing information. The only exception is the grid cells residing outside the national boundary of China, which are imputed by filling in zero values. While the zero values could influence the model performance, our analysis demonstrates the robustness of our results since the performance varies only moderately with the spatial coverage of each monitoring station. In addition, since some stations co-locate within the same grid cell, these stations correspond to identical input features. We group these stations using their average $PM_{2.5}$ concentration as the output variable, leaving 943 data points in the final data set for our study.

The ambient $PM_{2.5}$ concentration includes the influence of energy-related and nonenergy-related sources, including dust, residential and open burning of biomass. We exclude the $PM_{2.5}$ concentration caused by dust, open burning, and residential biomass burning from the $PM_{2.5}$ observation and modify the labels. The detailed methods are as follows:

1. The Ministry of Ecology and Environment (MEE) of China released the guidelines to quantify the influence of dust weather to $PM_{2.5}$ concentrations in China in 2017 (https://www.mee.gov.cn/gkml/hbb/bgt/201701/t20170106_394054.htm). According to the guidelines, the dust periods are determined by the following rules:

Start time:

(A) Urban PM_{10} hourly average concentration is higher than or equal to two times of the average PM_{10} concentration of the previous 6 hours and greater than 150 $\mu g/m^3$.

(B) $PM_{2.5}/PM_{10}$ ratio is less than or equal to 50% of the average of the previous 6 hours.

End time: The first hour when the hourly average PM_{10} concentration drops to a relative error of less than or equal to 10% of the average PM_{10} concentration in the first 6 hours of the dust period.

2. The contribution of residential biomass burning in China in 2015 was quantified by Zhao et al. with the Community Multiscale Air Quality (CMAQ) model.[33] The emission from residential biomass burning is usually of higher uncertainty. Zhao et al. merged the statistics data from multiple sources to get a reasonable estimate of biomass consumption in rural China.

3. The contribution of open burning in China in 2015 was quantified by Zheng et al. with the CMAQ model[34]. The activity data for biomass open burning is determined according to the method of Qiu et al.[72] They use the fire points data from several satellites to guarantee the locations of the open burning emission.

The $PM_{2.5}$ concentration from energy-related sources is calculated as follows:

$$c_{energy} = c_{no\_dust} \times (1 - r_{open} - r_{res\_biomass}) \qquad (1)$$

where c_{dust} is the $PM_{2.5}$ concentration filtered by the MEE guidelines; r_{open} and $r_{res\_biomass}$ are the relative contributions of open burning and residential biomass burning. In the studies of Zhao et al. and Zheng et al., the influence of dust was not considered. Therefore, we apply the relative contributions of these sources for the calculation.

The nonlinearity of chemical processes affects $PM_{2.5}$ pollution significantly. However, the nonlinearity mainly influences the formation of secondary aerosols but hardly works on primary PM[73]. The dust emission only consists of primary PM, and the emissions from open burning and residential burning of biomass are mostly primary PM. According to Zheng et al.[34], the SO_2 and NO_x emissions from these two sectors only took 1% and 5% of the anthropogenic emission in 2015 in China. Therefore, this estimation of the energy-related $PM_{2.5}$ concentration is rarely affected by the nonlinearity of atmospheric chemistry.

Data description

Table S1 presents the summary statistics of the variables in the full sample, the training, validation, and testing sets. The training, validation, and testing sets are created using stratified sampling. Specifically, China consists of seven regions based on the literature[30,32] and the official document[35], including (a) Bejing-Tianjin-Hebei (JJJ representing the first character of three provinces' short names) and surrounding cities (some cities in Shanxi, Shandong, and Henan province included), (b) Yangtze River Delta provinces (YRD), (c) Pearl River Delta provinces (PRD), (d) other East, (e) other Central, (f) West, and (g) Northeast. The full data set is first divided into seven pieces corresponding to the seven regions. Within each region, the data points are randomly divided into training, validation, and testing sets with a ratio of 3:1:1.

As shown in Table S1, the summary statistics of the three sub-samples (training, validation, and testing sets) are similar to the full sample in terms of the mean and standard

deviations, suggesting the representativeness of the three sub-samples. As a result, the parameters learned from the training set can represent the population characteristics, and the estimated average treatment effect (ATE) can be extrapolated toward the whole population.

Model design of ResCNN

Figure S1 illustrates the proposed ResCNN architecture. The top part is a standard AlexNet architecture using the normalized emissions from fossil fuel use and other geographical information as inputs. The bottom part adopts a linear specification using the average CO_2 emissions from energy consumption of each sector, the agricultural information (fertilizer use, livestock, and poultry production per unit of area), and the geographic information as inputs. The two parts are then combined by the $(\lambda, 1-\lambda)$ weighting. The formula of ResCNN is

$$y_n = \lambda\beta'[z_n, \bar{x}_{kn}] + (1-\lambda)f_c(x_{ijkn}) + \epsilon_n \qquad (2)$$

in which the z_n represents the control variables, the x terms represent the treatment variables, and ϵ_n represents the random noise. This ResCNN architecture is designed to resemble ResNet[42], which consists of a skip connection and a CNN model. However, the ResCNN model is even more general because of the $(\lambda, 1-\lambda)$ weighting with $\lambda \in [0,1]$. The weighting strategy enables ResCNN to flexibly resemble a linear model (as $\lambda \to 1$) or a CNN model (as $\lambda \to 0$). Intuitively, the ResCNN can also be seen as a hybrid model. Different from the typical hybrid model that combines physics and machine learning[70], the ResCNN model combines a reduced-form linear model with the highly nonlinear CNN structure. The λ term, which controls the balance of the linear and nonlinear components, can be tuned as a hyper-parameter using the validation set. This λ balancing strategy is highly effective, as demonstrated in Section Model performance below.

Causal interpretation in ResCNN

Although the CNN component $f_c(x_{ijkn})$ is highly nonlinear, the ResCNN adopts a semi-linear structure because the terms related to the control z_n, the treatment x_n, and the random noise ϵ_n are linearly added together in Equation 2. This innovative design facilitates the causal interpretation. In fact, the benefits in the high predictive power of the ResCNN can also directly translate to the causal model. While a simple linear model captures only the linear causal relationship, the CNN component captures the high-resolution nonlinear causal relationship. In addition, the ResCNN model is sample efficient with the regularization imposed by the hyper-parameter $(\lambda, 1-\lambda)$, implying that it can achieve high generalizability with a relatively small sample. The details of the ResCNN design and its benefits in statistical properties can be found in the related work[74]. Here we present a sketch proof of how the causal interpretation holds in the ResCNN.

Proposition 1 *Suppose the ResCNN model adopts a semi-linear structure as in Equation 2, and the conditional independence (CI) assumption $x_n \perp \epsilon_n | z_n$ holds, the marginal effect $\partial E[y_n|x_n, z_n]/\partial x_n$ has a causal interpretation.*

Proof. Let $\beta = [\beta_z, \beta_x]$ and $f_l(x_n) = \beta_x'\bar{x}_{kn}$. After simplifying the subscripts, we can rewrite Equation 2 as:

$$y_n = [\lambda\beta_z' z_n] + [\lambda f_l(x_n) + (1-\lambda)f_c(x_n)] + \epsilon_n \qquad (3)$$

Since the noise ϵ is linearly added in Equation 2, then

$$E[y_n|z_n, x_n] = \lambda\beta_z' z_n + \lambda f_l(x_n) + (1-\lambda)f_c(x_n) \qquad (4)$$

Using the causal notation from Angrist and Pischke[68], y_n takes the value of y_{0n} in the control group ($x_n = 0$) and that of y_{1n} in the treatment group ($x_n = 1$). We decompose the observed difference between the two groups into the average treatment effect (ATE) on the treated and the selection bias

$$\begin{aligned} &E[y_{1n}|z_n, x_n = 1] - E[y_{0n}|z_n, x_n = 0] \\ &= [E[y_{1n}|z_n, x_n = 1] - E[y_{0n}|z_n, x_n = 1]] \\ &+ [E[y_{0n}|z_n, x_n = 1] - E[y_{0n}|z_n, x_n = 0]] \end{aligned} \qquad (5)$$

Since $x_n \perp \epsilon_n | z_n$, then $\forall t(\cdot), t(x_n) \perp \epsilon_n | z_n$. Intuitively, when x_n and ϵ_n are conditionally independent, then any transformation of the two parts is still conditionally independent[71]. Let $t(x_n) = \lambda f_l(x_n) + (1-\lambda)f_c(x_n)$. Conditioning on z_n, we can obtain $y_{0n} \perp x_n | z_n$ and:

$$E[y_{0n}|z_n, x_n = 1] - E[y_{0n}|z_n, x_n = 0] = 0 \qquad (6)$$

Using the CI assumption $\{y_{0n}, y_{1n}\} \perp x_n | z_n$, the ATE on the treated is the same as ATE

$$\begin{aligned} &E[y_{1n}|z_n, x_n = 1] - E[y_{0n}|z_n, x_n = 1] \\ &\quad = E[y_{1n}|z_n, x_n] - E[y_{0n}|z_n, x_n] \end{aligned} \qquad (7)$$

Inserting Equations 6 and 7 into Equation 5, we can obtain:

$$\begin{aligned} &E[y_{1n}|z_n, x_n = 1] - E[y_{0n}|z_n, x_n = 0] \\ &\quad = E[y_{1n}|z_n, x_n] - E[y_{0n}|z_n, x_n] \end{aligned} \qquad (8)$$

Therefore, the regression equation of ResCNN can be used to compute the ATE, as long as the semi-linear structure and the CI assumption hold. More generally, when x_n is continuous, we can replace the left hand side of Equation 5 by the marginal effect $\partial E[y_n|x_n, z_n]/\partial x_n$, which also has a causal interpretation. □

Experiment design

We adopted a sequential hyper-parameter searching process by firstly searching for the hyper-parameters other than λ and secondly targeting λ for a thorough search. This sequential approach is adopted because the hyper-parameter space excluding λ is significantly larger than the λ space. The sequential approach disentangles the impacts of λ from others.

In the first round of hyper-parameter searching, we gradually reduce the hyper-parameter space from Table S3 to a pruned space in Table S4. Initially, the hyper-parameters are randomly chosen from Table S3 for training and comparison. After running a group of hyper-parameters,

some are pruned due to their relatively low performance. Finally, a grid search is completed to test all possible hyper-parameter combinations in the pruned hyper-parameter space.

In the second round, we search thoroughly for the possible λ values owing to their particular importance in the model design. Specifically, the λ values span the list of [0.0, 0.1, 0.2, 0.3, 0.4, 0.5, 0.7, 0.9, 0.95], which contains nine values, much more granular than any other hyper-parameter. Conditioning on each λ value, the models are repeatedly trained for 30 runs. This experiment design enables us to fairly compare the impacts of various λ values by averaging over many training runs and fixing a specific group of hyper-parameters.

Model performance

Figure S4 visualizes how the λ values influence the model performance in the training, validation, and testing sets. In Figure S4, the linear coefficient on the x-axis represents the λ factor applied to the linear model. Each point in the figure represents a model result, and a quadratic curve is used to fit through the model results to demonstrate the overall trend. The trend line suggests that optimal λ occurs between 0.1 and 0.3, which strikes a balance between high-quality predictive performance and model stability. As shown by Figure S4, the mean squared error (MSE) increases significantly in all three sets as the ResCNN approaches a pure linear model ($\lambda \rightarrow 1$). On the other side, the ResCNN model with $\lambda \in (0.1, 0.3)$ can slightly outperform a pure CNN model ($\lambda \rightarrow 0$). The optimum λ value roughly equals 0.2, which suggests that the ResCNN architecture approaches the best performance in both the validation and testing sets when it includes around 20% linear structure. Comparing the two endpoints ($\lambda = 0$ and $\lambda = 1$), we find that the CNN model demonstrates a significantly higher performance than the linear model, consistent with the recent machine learning literature.

Table S2 compares the model performance of the ResCNN, two CTMs, the linear regression, and the multi-task ResCNN results on the testing set. The two CTMs stem from the updated version of the Air Pollution Emission Experiments and Policy analysis model (AP2/AP3)[17] and the Intervention Model for Air Pollution (InMAP)[19]. The linear regression is incorporated since it is an important benchmark for the causal discussion, and it functions as a specific case of the ResCNN architecture. Multi-task learning is an important machine learning approach where the six pollutants ($PM_{2.5}$, PM_{10}, SO_2, NO_2, CO, and O_3) are trained together instead of training only for $PM_{2.5}$. Table S2 illustrates the superior predictive performance of the ResCNN model over the two CTMs and the linear regression. The performance of the uni-task and the multi-task ResCNN is highly comparable across all five metrics. This similar performance is reasonable since multi-task learning can improve the performance only when the tasks provide positive transferable meta-information[75]. When other target variables are a mixture of positively and negatively transferable tasks, the uni-task and multi-task learning results can be similar.

Figure S1.

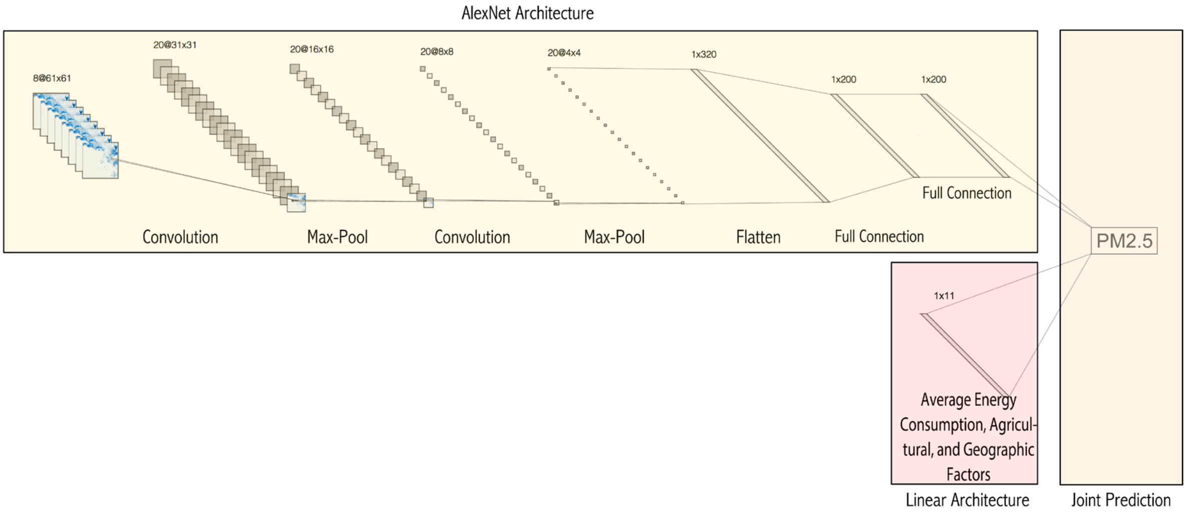

Figure S1. Architecture of the ResCNN framework, Related to Figure 1.

Figure S2.

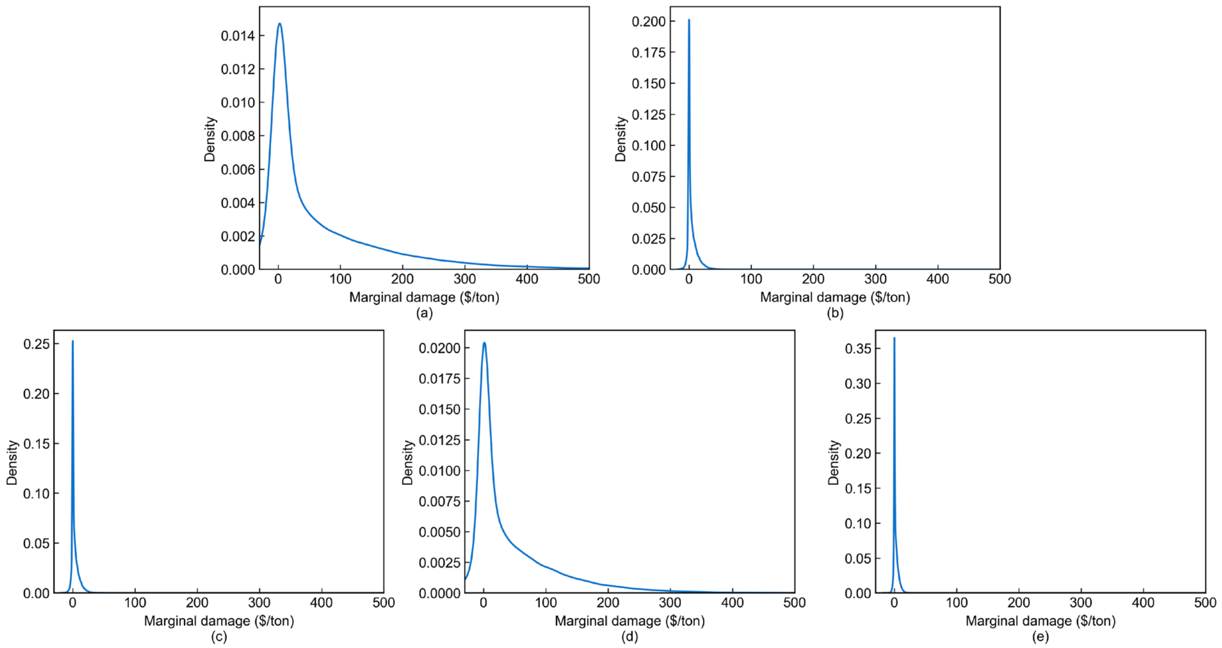

Figure S2. The distribution of marginal health damages attributable to an additional ton of CO_2 emissions: (a) rural and residential coal use, (b) coal use in the industry sector, (c) oil use in the industry sector, (d) coal use in the service sector, and (e) oil use in the transportation sector, Related to the Health co-benefits estimations section.

Figure S3.

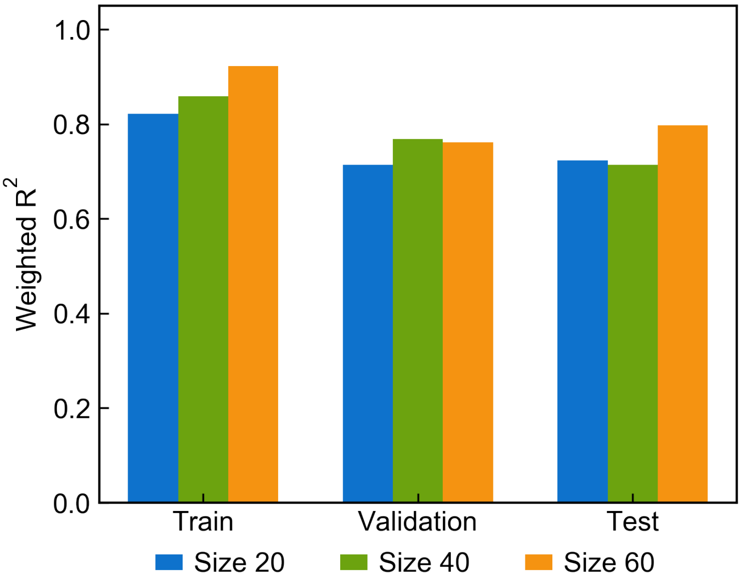

Figure S3. Prediction accuracy (measured by weighted R^2 on the train, validation, and test data sets) by models with different sizes of input area, Related to the Robustness analysis section.

Figure S4.

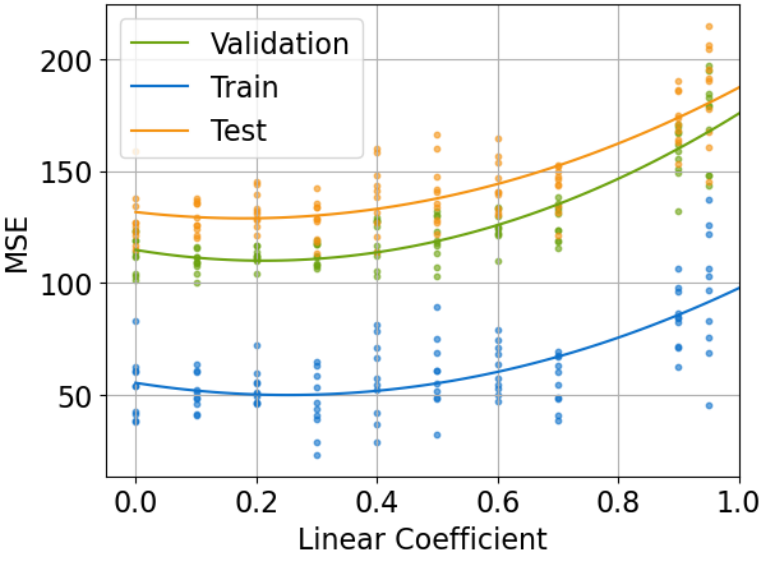

Figure S4. Prediction accuracy and λ values for predicting $PM_{2.5}$ (measured by weighted MSE on the train, validation, and test data sets), Related to the Model performance section in the Supplemental information.

Table S1. Data summary, Related to structure and performance of the Machine-learning framework section.

Variable	Mean				Standard Deviation				Data source
	All	Train	Validation	Test	All	Train	Validation	Test	
CO_2 from rural and residential coal (10 kton)	0.42	0.41	0.44	0.43	0.38	0.38	0.39	0.38	CHRED [a]
CO_2 from industrial coal (10 kton)	14.97	14.79	15.58	14.92	11.33	11.17	11.71	11.39	CHRED [a]
CO_2 from industrial oil (10 kton)	1.66	1.68	1.64	1.59	1.21	1.22	1.26	1.14	CHRED [a]
CO_2 from service sector coal (10 kton)	0.23	0.23	0.25	0.24	0.20	0.20	0.20	0.20	CHRED [a]
CO_2 from road transportation oil (10 kton)	1.97	1.96	2.02	1.94	1.18	1.20	1.21	1.12	CHRED [a]
Altitude (m)	0.07	0.07	0.07	0.07	0.08	0.08	0.08	0.07	CHRED [a]
Temperature (0.1℃)	0.88	0.89	0.89	0.85	0.49	0.49	0.50	0.49	CHRED [a]
Rain (0.1 mm)	0.01	0.01	0.01	0.01	0.00	0.00	0.00	0.00	CHRED [a]
Fertilizer per area (ton/km^2)	0.18	0.17	0.20	0.20	0.26	0.19	0.39	0.27	MSY [b]
Livestock per area (10,000/km^2)	0.21	0.21	0.22	0.23	0.25	0.24	0.16	0.36	MSY [b]
Poultry per area (10,000/km^2)	2.02	1.96	2.15	2.07	2.16	2.05	2.32	2.32	MSY [b]
Energy-related $PM_{2.5}$ (μg/m^3)	40.52	40.57	40.84	40.05	14.54	14.74	13.94	14.63	NAQDP [c]
PM_{10} (μg/m^3)	87.45	86.30	89.35	89.02	33.99	33.36	34.84	34.86	NAQDP [c]
SO_2 (μg/m^3)	25.10	25.26	24.59	25.12	15.82	16.05	15.47	15.44	NAQDP [c]
NO_2 (μg/m^3)	30.99	30.58	30.88	32.34	12.46	12.51	12.25	12.42	NAQDP [c]
CO (mg/m^3)	1.07	1.07	1.07	1.08	0.38	0.39	0.38	0.37	NAQDP [c]
O_3 (μg/m^3)	85.94	84.77	88.87	86.52	17.33	17.19	17.91	16.75	NAQDP [c]

[a] CHRED: China high-resolution emission database;
[b] MSY: Municipal statistical yearbooks;
[c] NAQDP: National air quality disclosure portal.

Table S2. Comparison of model performance (weighted by the population of each observation), Related to the Machine-learning framework section.

Evaluation Metrics	Equations	This study (test data)	InMAP	AP2	Linear Regression	Multitask Learning
Mean fractional bias (MFB)	$\frac{1}{N}\sum_n w_n \frac{2(y_n - \hat{y}_n)}{y_n + \hat{y}_n}$	0.05	-0.06	N/A	-0.0008	0.02
Mean fractional error (MFE)	$\frac{1}{N}\sum_n w_n \frac{2\lvert y_n - \hat{y}_n\rvert}{y_n + \hat{y}_n}$	0.17	0.36	N/A	0.21	0.17
Mean proportional error (MPE)	$\frac{1}{N}\sum_n w_n \frac{\lvert y_n - \hat{y}_n\rvert}{\hat{y}_n}$	0.18	N/A	0.37	0.23	0.18
Correlation coefficient (ρ)	$\frac{\sum_n w_n(y_n\hat{y}_n) - \sum_n w_n y_n \sum_n w_n \hat{y}_n}{\sqrt{\sum_n w_n (y_n - \bar{y}_w)^2}\sqrt{\sum_n w_n (\hat{y}_n - \bar{\hat{y}}_w)^2}}$	0.86	0.74	0.62	0.75	0.85
R^2	$\frac{\sum_n w_n (y_n - \bar{y}_w)(\hat{y}_n - \bar{\hat{y}}_w)}{\sqrt{\sum_n w_n (y_n - \bar{y}_w)^2}\sqrt{\sum_n w_n (\hat{y}_n - \bar{\hat{y}}_w)^2}}$	0.71	0.13	N/A	0.56	0.70

Note: y_n is the observed $PM_{2.5}$ annual average concentration, and $\hat{y}_n$ is the predicted $PM_{2.5}$ annual average concentration by a model. w_n is the population weight. $\bar{y}_w$ is the weighted average of y_n and $\bar{\hat{y}}_w$ is the weighted average of $\hat{y}_n$.

Table S3. Hyper-parameter search space, Related to the Hyper-parameter searching and training section.

Hyperparameters	Equations
Number of iterations	[100, 300, 500]
Size of mini batches	[20, 50, 100, 200]
Number of convolutional layers	[1, 2, 3, 4, 5]
Number of filters in convolutional layers	[20, 50, 80, 100]
Kernel size in convolutional layers	[2, 3, 4, 5, 6]
Stride size in convolutional layers	[1, 2]
Kernel size in max pooling layers	[2, 3, 4, 5, 6]
Stride size in max pooling layers	[1, 2]
Existence of dropout layers	[True, False]
Dropout rates	[0.0, 0.1, 0.2, 0.5]
Batch normalization	[True, False]
Number of fully connected layers	[1, 2, 3]
Width of fully connected layers	[50, 100, 200]
Existence of augmentation	[True, False]
ϵ variance in image augmentation	[0.05, 0.1, 0.2]

Table S4. Pruned hyper-parameter search space, Related to the Hyper-parameter searching and training section.

Hyperparameters	Equations
Number of iterations	[500]
Size of mini batches	[200]
Number of convolutional layers	[1, 2]
Number of filters in convolutional layers	[20, 50, 80]
Kernel size in convolutional layers	[2, 3]
Stride size in convolutional layers	[2]
Kernel size in max pooling layers	[2]
Stride size in max pooling layers	[2]
Existence of dropout layers	[True, False]
Dropout rates	[0.1]
Batch normalization	[True, False]
Number of fully connected layers	[1, 2]
Width of fully connected layers	[200]
Existence of augmentation	[True, False]
ϵ variance in image augmentation	[0.05, 0.1]

REFERENCES

74. Qiu, X., Duan, L., Chai, F., Wang, S., Yu, Q., and Wang, S. (2016). Deriving high-resolution emission inventory of open biomass burning in China based on satellite observations. Environ. Sci. Technol. *50*, 11779–11786.

75. Ding, D., Xing, J., Wang, S., Dong, Z., Zhang, F., Liu, S., and Hao, J. (2022). Optimization of a NO_x and VOC cooperative control strategy based on clean air benefits. Environ. Sci. Technol. *56*, 739–749.

76. Bertsekas, D.P., and Tsitsiklis, J.N. (2000). Introduction to probability (Athena Scientinis).

77. Willard, J., Jia, X., Xu, S., Steinbach, M., and Kumar, V. (2020). Integrating physics-based modeling with machine learning: A survey. arXiv Prepr. 2003.04919.
